# Systematic mapping review of interventions to prevent blood loss, infection and relapse in orthognathic surgery

**DOI:** 10.4317/medoral.25530

**Published:** 2023-02-18

**Authors:** Josefina Bendersky, Macarena Uribe, Maximiliano Bravo, Juan Pablo Vargas, Julio Villanueva, Gerard Urrutia, Xavier Bonfill

**Affiliations:** 1 Iberoamerican Cochrane Center, Institut d'Recerca-Servei d'Epidemiologia Clínica i Salut Pública, Barcelona, Spain; 2 Universitat autónoma de Barcelona, Campus de la UAB, Barcelona, Spain; 3 Department of Oral and Maxillofacial Surgery and Cochrane Associated Center at Faculty of Dentistry, University of Chile, Santiago, Chile; 4 Oral and Maxillofacial Surgery Program, Universidad de los Andes, Santiago, Chile; 5 School of Dentistry, Faculty of Medicine, Pontifical Catholic University of Chile, Santiago, Chile; 6 Maxillofacial Surgery service, Hospital Cliìnico San Borja-Arriaraìn Clinical, Santiago, Chile; 7 Consorcio de Investigación Biomédica en Red de Epidemiología y Salud Pública (CIBERESP), Madrid, Spain

## Abstract

**Background:**

This systematic mapping review aims to identify, describe, and organize the currently available evidence in systematic reviews (SR) and primary studies regarding orthognathic surgery (OS) co-interventions and surgical modalities, focusing on the outcomes blood loss, infection and relapse.

**Material and Methods:**

A comprehensive search strategy was performed to identify all SRs, randomized controlled trials and observational studies that evaluate surgical modalities and perioperative co-interventions in OS that evaluate the outcomes blood loss, infection and relapse, regardless of language or publication date. Searches were conducted in MEDLINE, EMBASE, Epistemonikos, Lilacs, Web of Science, and CENTRAL. In addition, grey literature was screened.

**Results:**

27 SRs and 150 primary studies fulfilled the inclusion criteria, 91 from SRs, and 59 from our search strategy. Overall, the quality of the SRs was graded as “Critically low,” and only two SRs were rated as “High” quality. 11 PICO questions were extracted from SRs and 31 from primary studies, which focused on osteosynthesis methods, surgical cutting devices, use of antibiotics, and induced hypotension. In addition, evidence bubble maps for each outcome were created to analyze in a visual manner the existing evidence.

**Conclusions:**

Future primary and secondary high-quality research should be addressed focused on the eight knowledge gaps identified in this mapping review. We concluded that the evidence mapping approach is a practical methodology for organizing the current evidence and identifying knowledge gaps in OS, helping to reduce research waste and canalize future efforts in developing studies for unsolved questions.

** Key words:**Orthognathic surgery, mapping review, le fort, blood loss, infection, relapse.

## Introduction

Orthognathic surgery (OS), is frequently used to treat dentofacial deformities that affect 20% of the population (1), restoring anatomic and functional relationships (2). According to the American Association of Oral and Maxillofacial Surgeons (AAOMS), conditions with an indication for orthognathic surgery include anteroposterior, vertical or transverse discrepancies, asymmetries, dysfunctions, temporomandibular joint disorders, speech impairments and airway dysfunctions. An increase in the frequency of OS performed each year has been shown, reaching a total of 8755 OSs performed in the United States in 2007 (3).

The production of scientific literature in the field of OS has increased significantly, however, resolved research questions and specific topics continue to produce a high flow of both primary and secondary research, meanwhile unresolved questions are not being addressed by researchers.

Although OS is considered a safe procedure, a variety of complications exists, which alter in accordance to different surgical modalities and perioperative co-interventions applied for the optimization of surgical results (1,2). Most include blood loss, infection and relapse (4-6). The reason for blood loss is the extensive vascularization of the maxillofacial region and access difficulty in terms of cauterization or ligation of the vessels involved (7). Regarding post operative infection, the proportion of patients developing surgical site infection after OS is estimated to be about 7%. It can cause localized pain, swelling, surface redness (erythema), pus formation and restricted movement (8). Moreover, relapse of the surgical movement has been associated with planning errors, intraoperative difficulties, anatomical variations, or limitations in the postoperative orthodontics (4). Though reported complications are limited, their occurance may suppose a risk for permanent deficiencies, need for reoperations, and impact the patient’s quality of life.

To facilitate evidence-informed decision making by making evidence available and presenting it in an accessible format, and to guide future research on blood loss, infection and relapse in OS, it is crucial to identify and analyze all the available evidence regarding co-interventions and surgical modalities which have an effect on these complications. Thus, the emerging synthesis method of mapping reviews constitutes a valuable mechanism for this particular area (9,10). To our knowledge, this is the first evidence synthesis that scopes all the existing evidence regarding blood loss, infection and relapse in OS.

Evidence mapping provides an innovative, friendly and didactic approach that illustrates the existing evidence in extensive research areas (9,11), leading to systematic characterization of evidence, identification of knowledge gaps, and prioritization of new research questions (9-11). Therefore, it can be the first step to conduct future systematic reviews (SRs) or the framework to inform policy development (9-11). Moreover, it allows the identification of resolved research questions, avoiding unnecessary effort of researchers in developing primary studies or SRs in the subject.

This mapping aims to identify, describe, and organize the currently available evidence in SRs and primary studies regarding OS co-interventions and surgical modalities, focusing on the outcomes blood loss, infection and relapse. Moreover, it assesses the quality of the existing evidence present and its claimed effectiveness and unveiled all the clinical questions regarding this topic. This review facilitates the identification of research gaps, allowing for new studies to develop.

## Material and Methods

This work is part of a broader mapping review of OS published elsewhere (12). It was drafted using the Preferred Reporting Items for Systematic Reviews and Meta-analysis (PRISMA)-Extension for Scoping Reviews. It adheres to the Global Evidence Mapping Initiative methods (GEM) (13), incorporating the quality of the supporting evidence. All methods are specified a priori in a published protocol (14).

- Mapping boundaries and context

To frame this mapping review, a preliminary search was performed and maxillofacial surgeons experts in OS were consulted, to establish eligibility criteria for study inclusion.

- Eligibility criteria

The population, intervention, comparison, and outcomes (PICO) framework was used to guide the eligibility criteria.

Studies: Included were SRs, as well as RCTs, prospective and retrospective observational studies (case-control and cohort studies). Only studies published as full texts and publications ahead of print were included. Excluded were narrative reviews, case series, case reports, qualitative and cross-sectional studies.

Population. Adult and adolescent participants (aged over 10 years) undergoing OS were included. Syndromic patients were excluded.

Interventions: Perioperative co-intereventions and different surgical modalities of OS were considered. Studies regarding distraction osteogenesis and those which focus relied on orthodontic procedures or surgical planning were excluded.

Comparators: Different approaches to performing OS, different co-interventions, and use of placebo or no treatment (control group) associated with co-interventions were included. Excluded were studies with no comparison group or studies in which the comparison group did not undergo OS.

Outcomes: Studies that assessed blood loss, infection and relapse were included.

- Literature search

A literature search was conducted from inception to March 2021 in the following databases: MEDLINE (via PubMed), EMBASE (via OVID), Epistemonikos, Lilacs, Web of Science, and CENTRAL. The search strategy, adapted for each database considering differences in controlled vocabulary and syntax rules, is available elsewhere (12). No date or language restrictions were applied. Unpublished SRs were also searched in the PROSPERO protocol registration platform. A snowball approach was used for screening reference lists to identify potentially eligible studies. Although the protocol stated that a search would be conducted in ClinicalTrials.gov to detect ongoing primary studies, due to the large quantity of studies identified, it was decided not to include articles from this platform, as the benefits would have been marginal given the extensive data already compiled.

- Data collection and analysis

Two independent reviewers performed a title/abstract screening for results obtained from the search, followed by a full-text screening, independently and in duplicate. Reasons for exclusion were recorded. Any disagreements were resolved by consensus or, if necessary, by a third author. As recommended in the Cochrane Handbook for Systematic Reviews of Interventions, the selection process was documented in sufficient detail to generate a PRISMA flowchart.

- Data extraction and management

Data was extracted and tabulated by one author, using a previously piloted data extraction form, and was double-checked by a content expert in the subject. The following information was extracted from each SR: search date, year of publication, country, objective, number of primary studies included, number of participants, population, co-interventions, comparisons, and outcomes measured. Research PICO questions of the SRs were also identified. For primary studies not included in any identified SRs, the following data were extracted: year of publication, study design, objective, number of patients included, population, interventions, comparisons, and outcomes. Author’s conclusions were recorded and classified for descriptive purposes as "beneficial," "probably beneficial," "no differential effect," "harmful," or "inconclusive". Supplement 1 indicates the criteria that was used for this categorization.

- Methodological quality evaluation

Methodological quality was independently evaluated for all the included SRs by two authors. Disagreements were resolved by consensus, or if necessary, by a third author. The AMSTAR-2 instrument (15) was used to evaluate the included SRs, which critically appraises SRs in 16 domains. The overall rating was based on weaknesses in critical domains, namely, items 2, 3, 7, 9, 11, 13, and 15. Global confidence in SR results - used for the mapping diagrams - was rated in the following four quality categories: critically low (more than one critical flaw), low (one critical flaw), moderate (more than one non-critical flaw), and high (no flaw or one non-critical flaw). The included primary studies were not critically appraised since they were included to identify knowledge gaps rather than to inform clinical or policy decisions.

- Data synthesis and analysis

Results, summarized in tabular formats, describe the included study characteristics and all the identified PICO questions. An evidence matrix to link primary studies with their SRs was created using the Epistemonikos platform to identify primary studies not included in the SRs. Evidence bubble maps were also created.

## Results

- Search results

**Figure 1:**
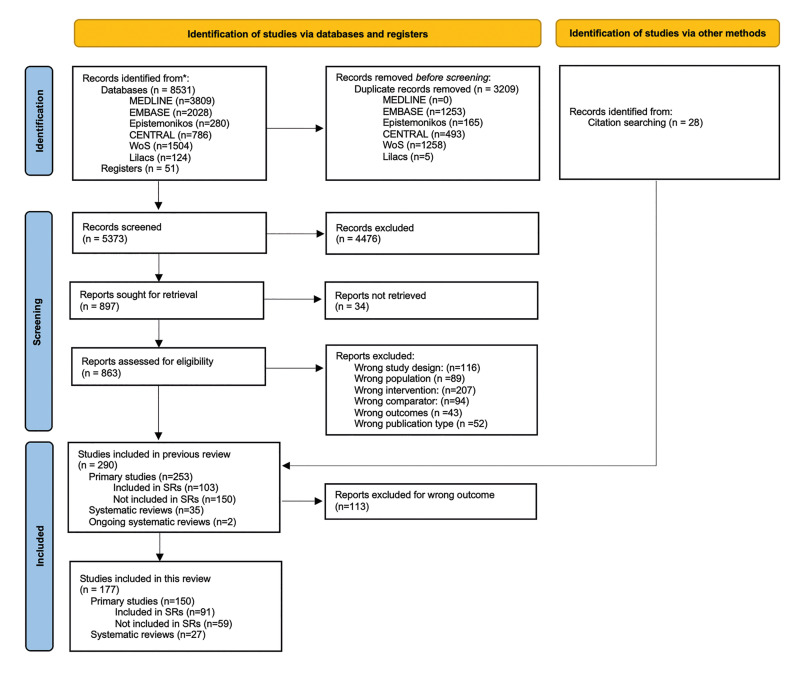
PRISMA flow chart.

8531 records were obtained. In the title/abstact phase, 5373 records were screened. Of the latter, 4476 records were excluded. 863 articles were assessed by full-text reading, resulting in 262 included articles. Reasons for exclusion at detailed in Fig. 1. Moreover, throughout hand-searching, 28 articles, 1 SR, and 27 primary studies were identified and included in the review. A total of 290 studies were included in the general mapping review. For this specific mapping review, 113 studies were excluded for not reporting on the three outcomes of interest (bloos loss, infection, relapse). Thus, a final total of 177 studies were included, 27 SRs and 150 primary studies (91 included and 59 not included in the SRs). Kappa value was 0.81, thus considered excellent agreement. A PRISMA flowchart details this process in Fig. 1.

- Study characteristics

A general description of the study characteristics, including design, aim, population, interventions, comparators, outcomes, and number of primary studies and participants is presented in Supplement 2.

- Study designs

Of the 177 included articles, 27 (27/177; 15.3%) referred to SRs and 150 (150/177; 84.7%) to primary studies. Of the latter, 91 (91/177; 51.4%) were studies included in the identified SRs. The remaining 59 (59/177; 33.3%), identified through the Covidence platform screening, were not included in the SRs. Their designs were as follows: prospective cohort (6/59; 10.2%), retrospective cohort (25/59; 42.4%), and RCT (28/59; 47.5%). Supplement 3 organizes included studies according to their design and outcome assessed.

- Participant characteristics

Age: Seven SRs (25.9%) included only adult populations, two (7.4%) included patients of all ages, and 18 (66.7%) did not mention age.

Regarding the age of participants in the 59 primary studies not included in SRs, 15 studies (25.4%) included adolescent and adult patients, 34 studies (57.6%) included only results for adults >16 years, one study (1.7%) included participants of all ages, and 9 studies (15.3%) did not specify age.

Deformity: The type of deformity of participants was recorded in a simplified manner, grouped as either skeletal class II (mandibular retrognathism, mandibular hypoplasia, maxillary prognathism, and maxillary hyperplasia) or class III (mandibular prognathism, mandibular hyperplasia, maxillary retrognathism, and maxillary hypoplasia). Facial asymmetries in participants was also documented. Of the 27 SRs, one (3.7%) included patients with skeletal class III diagnosis and one (3.7%) with skeletal class II, while the remaining 25 (92.6%) did not specify skeletal classes as inclusion or exclusion criteria. Of the 59 primary studies, 13 (22%) included patients with skeletal class III diagnoses (one with facial asymmetry), three (5.1%) included patients with skeletal class II and class III diagnoses, 13 (22%) included results for patients with skeletal class II diagnoses, and 30 (50.8%) did not specify deformity inclusion criteria.

OS type: As for the type of surgery performed, one of the 27 SRs (3.7%) included patients undergoing bimaxillary surgery, four (14.8%) undergoing mandibular surgery, one (3.7%) maxillary surgery, one (3.7%) genioplasty, and 20 (74.1%) did not specify the type of intervention. Of the 59 primary studies, 13 (22%) included patients undergoing bimaxillary OS, four (6.8%) bimaxillary and genioplasty, one (1.7%) genioplasty alone, one (1.7%) genioplasty and other orthognathic procedures, 17 (28.8%) mandibular surgery, nine (15.3%) maxillary surgery, 10 (16.9%) a combination of the procedures mentioned above and four (6.8%) did not specify a type of orthognathic procedure.

- Methodological quality assessment

Based on AMSTAR-2 scores, 16 SRs (16/27; 59.3%) were rated as “Critically low”, four as “Low” (4/27; 14.8%), five as “Moderate” (5/27; 18.5%) and two were rated as “High” methodological quality (2/27; 7.4%) (see Supplement 4) The SRs were mainly downgraded as the authors did not state the development of a pre-existing protocol (16/27; 59.3%), nor did they explain their selection of study designs for inclusion in the review (18/27; 66.7%). Moreover, sources of funding of primary studies were not clearly stated (25/27; 92.6%), and the authors did not perform a study selection (12/27; 44.4%) and data extraction (12/27; 44.4%) in duplicate.

The best-rated items were attributed to search strategy, description of included studies in adequate detail, and investigation for publication bias.

- PICO questions

11 PICO questions were retrieved from the SRs and 31 from the primary studies (Supplement 5, Suplement 6). For the SRs, the PICO questions were grouped according to the OS type: all types of surgery, mandibular surgery, maxillary surgery, or genioplasty. For practical matters regarding classification, primary study questions were grouped by intervention, leaving aside their population.

- Evidence matrix

The identified SRs were linked with their included primary studies which fulfilled our selection criteria, to create an evidence matrix with the platform Epistemonikos. It is worth mentioning that one SR was not included in the matrix, as it was an update to an already included Cochrane review. Furthermore, one SR could not be added because of technical issues of the platform. The complete matrix is available in the following link: http://www.epistemonikos.org/matrixes/61f044727db23a0765984cfb

- Evidence bubble maps

**Figure 2:**
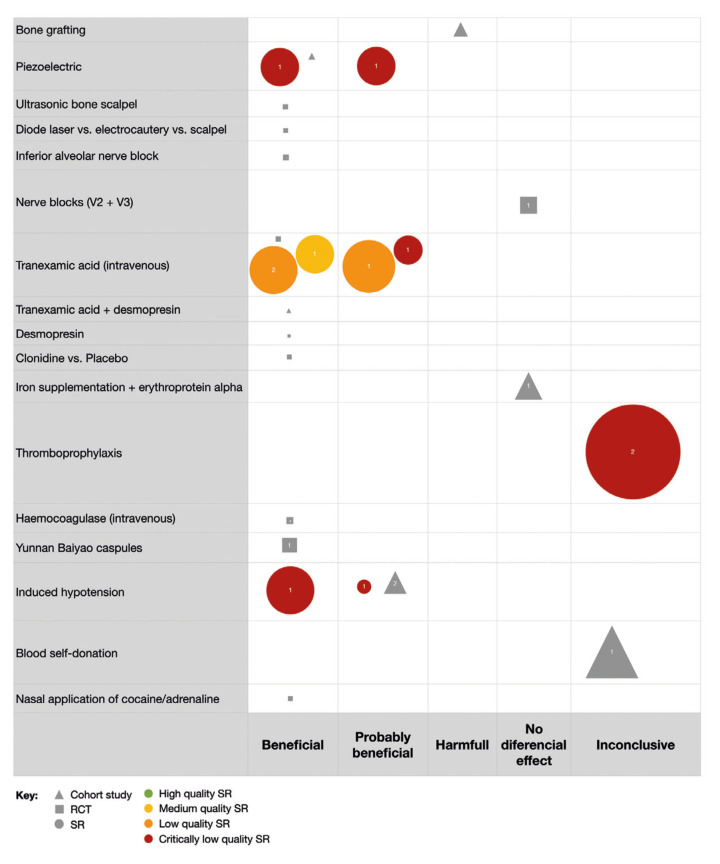
Bubble map for blood loss in OS.

**Figure 3:**
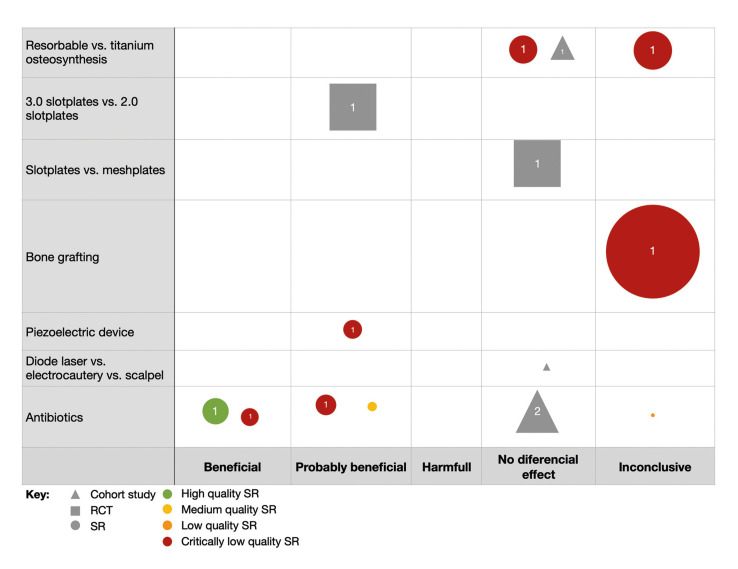
Bubble map for infection in OS.

**Figure 4:**
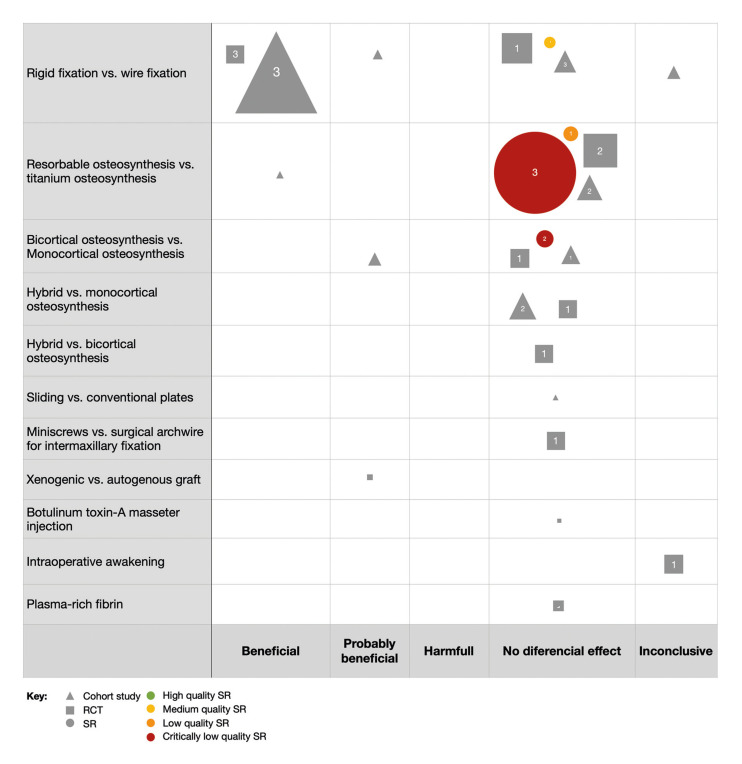
Bubble map for relapse in OS.

Evidence bubble maps were created per outcome to graphically depict the available evidence (Fig. 2, Fig. 3, Fig. 4).

All interventions reviewed in the included studies are listed in rows, and the direction of the results are listed in columns. Symbols indicate the study design (circles for SRs, squares for RCTs, and triangles for observational studies), and bubble size represents the number of primary studies included in the SRs or the number of participants included in the primary studies. A number positioned over each bubble indicates the number of studies. Colors indicate confidence in SR findings (based on the AMSTAR-2 evaluation). The primary studies included in the maps are those not included in any of the identified SRs.

- Blood loss

The outcome blood loss was evaluated by 11 SRs (11/35; 31.4%) and reported by 27 primary studies (27/150; 18%), 16 RCTs (16/150; 10.70%), seven retrospective cohort (7/150, 4.7%) and three prospective cohort (3/150; 2%) (Fig. 2).

Co-interventions which demonstrated a beneficial/probably beneficial effect consisted in IV administration of tranexamic acid (five SR of medium, low and critically low quality, *n*=1621; one RCT, *n*=36), induced hypotension (two low quality SR, *n*=560; two cohort studies, *n*=150), nasal application of cocaine/adrenaline (one RCT, *n*=30), administration of Yunnan Baiyao capsules (one RCT, *n*=95), IV haemocoagulase (one RCT, *n*=46) and clonidine (one RCT, *n*=30). On the other hand, the surgical modalities that showed a beneficial/probably beneficial reduction in blood loss were the use of a piezoelectric device (two critically low-quality SRs, *n*=1680; one cohort study, *n*=44), ultrasonic bone scalpel (one RCT, *n*=34), the use of diode laser compared to electrocautery and scalpel (one RCT, *n*=30), and blockage of inferior alveolar nerve with local anesthesia (one RCT, *n*=38). Only one observational study assessed blood loss in bone grafting, and concluded its use increased blood loss. Moreover, nerve blocks were performed in the maxillary and mandibular ramus of the trigeminal nerve (one observational study, *n*=110), and iron plus erythropoietin alpha supplementation (one RCT, *n*=178) were considered as having no differential effect on blood loss. Inconclusive results were reported for the administration of thromboprophylaxis (two SR of critically low quality, *n*=9707) and preoperative blood self-donation 7 days before surgery (one observational study, *n*=345). Note that studies that compared different anesthetic regimens were grouped in the co-interventions bubble map, as analysis of this issue was beyond the scope of this mapping review.

- Infection

Postoperative infection was assessed by nine SRs (9/35; 25.7%) and seven primary studies (7/150; 4.7%) not included in the mentioned SRs: four RCTs (4/150; 2.7%), one prospective cohort (1/150; 0.7%) and three retrospective cohort (3/150; 2%) (Fig. 3).

The use of piezoelectric, compared to conventional rotatory devices, demonstrated a beneficial/probably beneficial effect on infection (one critically low SR, *n*=996). Regarding fixation method, the use of Slotplates 3.0 compared to Slotplates 2.0 (one RCT, *n*=186) showed a beneficial effect on infection. However, Slotplates compared to Meshplates as a fixation method showed no differential effect in a cohort study of 190 participants. Furthermore, studies evaluating resorbable osteosynthesis compared to titanium osteosynthesis acquired the same conclusion (one critically low-quality SR, *n*=1384, one RCT, *n*=101), whereas other SR from the same authors showed inconclusive results (*n*=1092). Finally, studies reporting infection for the use of antibiotics in the perioperative surgical period had heterogeneous conclusions, from beneficial/probably beneficial (one high quality SR, *n*=788; two critically low-quality SR, *n*=698; one medium quality SR, *n*=452), no differential effect (two cohort studies, *n*=178) to inconclusive (one low quality SR, *n*=132).

- Relapse

Nine SRs (9/35; 25.7%) assessed the outcome relapse, as well as four prospective (4/150; 2.7%), 14 retrospective cohort studies (14/150; 9.3) and 10 RCTs (10/150; 6.7) (Fig. 4).

The majority of the studies concluded that co-interventions and surgical modalities had no differential effect on relapse. This was the case for resorbable compared to titanium osteosynthesis (three critically low quality SRs, *n*=3823; one low quality SR, *n*=420, one moderate quality SR, *n*=120, two RCTs, *n*=141, two cohort, *n*=108, one ongoing SR), bicortical compared to monocortical osteosynthesis (two critically low quality SRs, *n*=513; one RCT, *n*=76; two cohort, *n*=78), hybrid compared to monocortical osteosynthesis (two cohort, *n*=114; one RCT, *n*=76) and to bicortical osteosynthesis (one RCT, *n*=76), sliding plates (one cohort, *n*=23), miniscrews (one cohort, *n*=74), injection of Botulinum toxin-A into the masseter muscle (one cohort, *n*=16) and the use of platelet-rich fibrin (one RCT, *n*=44). Heterogeneous conclusions were observed in rigid compared to the wire fixation method. A recent SR of moderate quality (*n*=187) and primary studies (one RCT, *n*=127; three cohort, *n*=92) concluded no differential effect on the use of rigid fixation on relapse. Nevertheless, other primary studies of considerable population size, reported beneficial/probably beneficial (three RCT, *n*=345; four cohort, *n*=105) and inconclusive findings (one cohort, *n*=54).

- Research gaps

Supplement 7 lists eight identified research gaps that remain unanswered by evidence synthesis, either because they were identified in primary studies and not in SRs, or because of the low or critically low quality of the existing evidence.

## Discussion

This review aimed to identify, describe, and organize currently available evidence in SRs and primary studies regarding OS co-interventions, surgical modalities, focusing on the outcomes blood loss, infection and relapse. We therefore performed a systematic mapping review, a relatively new methodological approach focused on the identification of knowledge gaps, prioritization of future research needs in broad fields and displaying the obtained results in a user-friendly format (9). Our mapping review was based on 27 published SRs that included 91 individual studies, and an additional 59 primary studies that were not included in the identified SRs.

Methodological quality assessment using the AMSTAR-2 tool classified most of the SRs as critically low quality (16/27; 59.3%), and only identified two SRs as high quality. Domains that should be improved in future reviews are primarily: [1] development of an a-priori protocol to state that the methods were established previous to the conduct of the SR; [2] an explanation of the selection of study designs for inclusion in the review; [3] reporting the funding sources of primary studies and [4] performing the study selection and data extraction in duplicate. Although quality assessment is not a requirement of mapping reviews, we considered it an advantage in the terms of drawing more accurate conclusions.

Blood loss: Most of the assessed interventions were found to be beneficial for blood loss. For instance, intravenous administration of tranexamic acid has been widely investigated and found favorable for this outcome. Due to the presence of a medium-quality SR and the fact that beneficial conclusions were drawn from all studies, no future efforts should be addressed at answering this question. Alternatively, even though the use of piezoelectric devices and induced hypotension have also shown beneficial results in SRs with large number of studies, high-quality SRs should be developed to confirm these statements. Unfortunately, no recent RCTs have been developed on these topics. Moreover, the effect on blood loss in interventions such as administration of Yunnan Baiyao capsules, haemocoagulase, nasal application of cocaine, and inferior alveolar nerve blocks should be addressed in future primary studies, as the existing data shows beneficial results on this outcome.

Infection: The outcome of postoperative infection was assessed in fewer interventions. The use of piezoelectric device appears to be probably beneficial for preventing infection, although assessed by a critically low-quality SR (16). No new primary studies have been developed on this topic.

High variability was found in the conclusions of SRs which evaluated the use of antibiotics in OS, ranging from beneficial, probably beneficial, inconclusive, to no differential effect. This phenomenon might be explained because of the high heterogeneity of primary studies, which compare different antibiotics and regimens. Moreover, Brignardello-Petersen *et al*. (8), the only SR evaluating perioperative antibiotics with a high methodological quality, reported moderate and low confidence in their results, mainly because of unclear risk of bias in allocation concealment and blinding phases of the included RCTs. Thus, future research should focus on high-quality RCTs to determine the real effect of perioperative antibiotics in OS.

Relapse: Different osteosynthesis methods, such as resorbable compared to titanium and bicortical compared to monocortical fixation, did not seem to have a differential effect on postoperative relapse. Only one study for resorbable (*n*=30) and one for bicortical osteosynthesis (*n*=50) reported beneficial and probably beneficial results, respectively. Nevertheless, these correspond to cohort designs with a low number of patients. For rigid versus wire fixation, contradictory results were found. One SR reported no differential effect of both techniques (17), whereas three RCTs (18-20) and four cohort (21-24) refer a beneficial or probable beneficial effect for relapse. It is worth mentioning that the SR only included patients undergoing genioplasty procedures. Therefore, this mapping suggests that future evidence synthesis should be carried out on the effect of rigid compared to wire fixation in orthognathic patients undergoing mandibular, maxillary, or bimaxillary procedures. Nevertheless, as wire fixation requires a period of maxillomandibular fixation, over the past years its use has been widely replaced for rigid fixation regarding practical reasons. Hence, addressing this research gap is not a priority. Moreover, the effects of hybrid fixation (use of bicortical and monocortical screws) on relapse have only been reported by two cohort (25,26) and one RCT (27). No SRs have been carried out on this topic due to the low number of primary studies. Yet, no superior efficacy has been shown.

To our knowledge, this is the first mapping review performed for blood loss, infection and relapse in OS. However, a broader mapping review aimed at identifying knowledge gaps within the field of oral and maxillofacial surgery, was published in 2017 (28). This mapping included SRs from several domains, including reconstructive surgery, surgical tooth removals, tumors, orofacial infections, maxillofacial and dental trauma, OS, among others. Of the nine SRs included in the mentioned mapping, two were also present in our review (29,30). The seven excluded SRs did not fulfill our selection criteria: reports on orthopedic surgery (31), orthodontic SR, no control group (32,33), postoperative treatment for nerve injuries (34) and wrong outcomes (35-37). Additionally, no graphical representation of their results was illustrated. The information obtained from our evidence maps enabled us to identify several OS research gaps (summarized in Supplement 7) that should help direct future secondary research efforts in resolving unresolved questions regarding OS.

- Strengths

We highlight the sensitive search strategy implemented without language or date restrictions. Thus, relevant studies were unlikely missed. Furthermore, the evidence matrix allowed us to identify primary studies that responded to our inclusion criteria but were not included in the identified SRs. Therefore, knowledge gaps in topics covered by primary studies without a synthesis of the results performed by a SR were identified. Moreover, selection process and quality assessment were performed independently and in duplicate. Likewise, extraction data was checked by a content expert in the subject, thus, there is confidence in the results. In addition, to organize the obtained information, we used the PICO format to arrange the results, ensuring a simplified yet thorough approach of displaying the evidence on procedures and co-interventions in OS. Even though the evidence mapping methodology does not include a quality assessment of SRs (10), we performed this analysis. This advantage will enable the identification of topics in which new high-quality SRs should be performed to answer clinical questions. Moreover, it will help stakeholders to identify the quality of evidence on which clinical decisions are based. Finally, the graphical display developed for this review provides a user-friendly, visual approach to identify all the existing evidence of this topic and its quality.

- Limitations

First, conference abstracts and primary studies registries were not included in this mapping due to the extended volume of studies found. Nevertheless, we are confident that their incorporation would have not substantially modified the obtained results due to the large quantity of information compiled. Also, the imminent risk of a possible publication bias is present, which would have limited the inclusion of studies that were not published because of their non-favorable results. Finally, this methodology only organizes and describes evidence as reported by the original authors of studies, describing results as beneficial even if they are based on low-quality evidence. Therefore, this is not an appropriate tool for healthcare clinical decision-making.

## Conclusions

This evidence map was developed on SRs, RCTs, and observational studies, which assessed blood loss, infection and relapse in OS. 11 PICOs were identified in the SRs and 31 from primary studies, which primarily addressed the effects of fixation methods, surgical cutting devices, antibiotics, and induced hypotension. The methodological quality of most included SRs in this mapping review was rated as “Critically low.” Moreover, bubble maps allowed the identification of eight research gaps, including: to evaluate the effect of thromboprophylaxis and the efficacy of piezoelectric in blood loss, to compare the efficacy of resorbable vs. titanium fixation in infection, to assess the use of bone graftings in infection, to compare the use of rigid vs. wire fixation and bicortical vs. monocortical osteosynthesis in relapse, and to assess the efficacy of hybrid fixation in relapse in patients undergoing orthognathic surgery. This research gaps should be addressed in future high-quality research, both primary and secondary.
